# BUB1 promotes lung adenocarcinoma progression by regulating STAT3/GPX4-mediated ferroptosis

**DOI:** 10.3389/fonc.2025.1745238

**Published:** 2026-02-02

**Authors:** Xiaocong Mo, Ying Liu, Yu Wang, Xiaofen Pan, Mengyuan Zhu, Jiehao Liao, Minling Liu, Tingwei Li, Xueying Li, Shuo Fang, Bo Wang

**Affiliations:** 1 Department of Oncology, The Seventh Affiliated Hospital, Sun Yat-Sen University, Shenzhen, Guangdong, China; 2 Department of Oncology, The First Affiliated Hospital of Jinan University, Jinan University, Guangzhou, Guangdong, China; 3 Department of Geriatrics, Chongqing General Hospital, Chongqing University, Chongqing, China; 4 National Clinical Research Center for Digestive Diseases, Xijing Hospital of Digestive Diseases, Fourth Military Medical University, Xi’an, Shaanxi, China

**Keywords:** BUB1, lung adenocarcinoma, ferroptosis, STAT3, GPX4

## Abstract

**Introduction:**

Lung adenocarcinoma (LUAD) is the leading cause of cancer-related mortality worldwide, but its therapeutic efficacy remains suboptimal. This study explores the functional role and underlying mechanism of BUB1 in LUAD.

**Methods:**

*In vitro*, BUB1 knockdown (si-BUB1) in A549/H1299 cells was performed, and effects were assessed. The ferroptosis inhibitor Fer-1 was used. Mechanistically, the role of the STAT3/GPX4 axis was investigated through overexpression experiments. *In vivo*, xenograft models were used.

**Results:**

Bioinformatics analysis highlighted a significant upregulation of BUB1 in LUAD tissues, with elevated expression levels correlated with reduced disease-free survival (DFS) and overall survival (OS). BUB1 knockdown markedly suppressed cell proliferation, migration, and invasion, while concurrently inducing ferroptosis. This was evidenced by typical mitochondrial morphological changes (shrinkage, increased membrane density, reduced cristae), altered ferroptosis-related markers (decreased FTH1/SLC7A11, increased COX2), elevated Fe²^+^/MDA levels and reduced GSH activity, which could be reversed by Fer-1. BUB1 silencing suppressed the expression and phosphorylation of STAT3, thereby downregulating the transcription of GPX4. Overexpression of STAT3 and GPX4 partially reversed the inhibitory effects of BUB1 knockdown on LUAD cell malignancy and abrogated the ferroptosis induced by BUB1 silencing. *In vivo*, xenograft models further validated that BUB1 silencing significantly reduces tumor volume, accompanied by modulation of ferroptosis-related genes in tumor tissues.

**Discussion:**

Collectively, our findings identify BUB1 as a novel prognostic biomarker and therapeutic target for LUAD, revealing a new regulatory mechanism by which BUB1 promotes LUAD progression through the activation of the STAT3/GPX4 axis to suppress ferroptosis.

## Introduction

Lung adenocarcinoma (LUAD), the most common histological subtype of non-small cell lung cancer (NSCLC), remains the foremost contributor to cancer-related deaths worldwide (1). Despite significant advancements in therapeutic modalities, including targeted therapy and immunotherapy, the prognosis for LUAD patients remains dismal. This is primarily due to the absence of specific molecular targets and an incomplete understanding of the underlying pathogenic mechanisms (2). Therefore, identifying novel oncogenic drivers and elucidating their regulatory networks is essential for developing more efficacious diagnostic and therapeutic approaches for LUAD.

Budding uninhibited by benzimidazoles 1 (BUB1), a core component of the spindle assembly checkpoint, is indispensable for ensuring accurate chromosome segregation during mitosis (3). Dysregulation of BUB1 has been implicated in the pathogenesis and progression of multiple malignancies, including breast cancer, colorectal cancer and pancreatic cancer (4–6). In these tumors, overexpression of BUB1 contributes to genomic instability, cell cycle dysregulation, and enhanced tumor cell proliferation, ultimately correlating with adverse clinical outcomes. Nevertheless, the clinical significance, biological functions, and molecular mechanisms of BUB1 in LUAD remain largely undefined, representing a critical knowledge gap in current lung cancer research.

Ferroptosis, a recently characterized form of regulated cell death distinct from apoptosis, necrosis, and autophagy, is characterized by iron-dependent lipid peroxidation and the disruption of cellular redox homeostasis (7). This process is tightly regulated by a series of key molecules, including glutathione peroxidase 4 (GPX4), which detoxifies lipid peroxides to maintain the integrity of the lipid bilayer (8). Dysregulation of ferroptosis has been implicated in tumor progression, and inducing ferroptosis has emerged as a promising therapeutic strategy for various cancers, including LUAD (9). Notably, LUAD cells often exhibit dysregulated ferroptosis-related pathways, contributing to tumorigenesis and therapeutic resistance. However, the relationship between BUB1 and ferroptosis in LUAD has not been previously explored.

Signal transducer and activator of transcription 3 (STAT3), a central mediator of the JAK-STAT signaling pathway, is frequently hyperactivated in LUAD (10). Activated STAT3 promotes tumor progression by regulating the expression of genes involved in cell proliferation, survival, migration and metabolic reprogramming (11). Recent studies have demonstrated that STAT3 directly binds to the promoter region of GPX4 to upregulate its transcription, thereby suppressing ferroptosis (12–14). These suggest that the STAT3/GPX4 axis may serve as a critical regulatory pathway for ferroptosis in tumors. However, whether this axis is modulated by BUB1 to regulate LUAD progression and ferroptosis remains unknown.

In the present study, we hypothesized that BUB1 is aberrantly expressed in LUAD and contributes to tumor progression by regulating ferroptosis through the STAT3/GPX4 axis. To test this hypothesis, we systematically investigated the expression patterns and clinical significance of BUB1 in LUAD, evaluated its effects on the malignant phenotypes of LUAD cells and ferroptosis both *in vitro* and *in vivo*, and elucidated the underlying molecular mechanisms. Our findings aim to identify BUB1 as a candidate prognostic biomarker and therapeutic target for LUAD, while revealing a new regulatory network that links mitotic signaling to ferroptosis in lung cancer.

## Materials and methods

### Cell lines and grouping

Human LUAD cell lines (A549, H1299 and PC9) and normal lung epithelial cell line (HBE) were generously provided by Dr. Feng Ma. All cells were routinely cultured in RPMI-1640 (Gibco, Thermo Fisher Scientific, USA), supplemented with 10% fetal bovine serum (FBS, NEST Biotechnology) in culture flasks (SAINING, Biotechnology).

### RNA interference and plasmid transfection

Small interfering RNA (siRNA) targeting BUB1 (si-BUB1), non-targeting control siRNA (si-NC), shRNA targeting BUB1 (sh-BUB1), STAT3 overexpression plasmid (oe-STAT3), GPX4 overexpression plasmid (oe-GPX4) and empty vector were designed and synthesized by GenePharma (Shanghai, China). Cells were seeded into 6-well plates at 5×10^5^ cells/well and transfected with siRNAs or plasmids following the manufacturer’s protocol.

### CCK-8 assay

Cells were seeded into 96-well plates at 1×10^4^ cells/well. At 0–5 d post- transfection, 10 μL CCK-8 solution (Life-iLab, Shanghai, China) was added to each well and incubated for 2 h at 37 °C. Absorbance at 450 nm was measured using CYTATION 5 Reader (BioTek, USA).

### Wound healing assay

Confluent cells in 6-well plates were scratched with a sterile 200 μL pipette tip. After washing with PBS twice, cells were cultured in serum-free medium. Wound images were captured at 0 and 24 h using an inverted microscope (Olympus, Tokyo, Japan).

### Colony formation assay

Cells were seeded into 6-well plates at 500 cells/well and cultured for 14 days. Colonies were fixed with 4% paraformaldehyde for 30 min, stained with 0.1% crystal violet (Jiangsu Maige Biotechnology Co., Ltd.) for 15 min.

### Transwell assay

Transwell chambers (8 μm pore size, Corning, NY, USA) were pre-coated with Matrigel (BD Biosciences, USA) at 37 °C for 30 min. Cells (1×10^5^) in serum-free medium were added to the upper chamber, and medium containing 10% FBS was added to the lower chamber. After 24 h incubation, non-invaded cells on the upper membrane surface were removed with a cotton swab. Invaded cells were fixed, stained and counted in five random fields under a microscope.

### Reverse Transcription-Quantitative Polymerase Chain Reaction

Total RNA was extracted from LUAD cells and tumor tissues using TRIzol reagent (Invitrogen, USA). RNA concentration and purity were determined with a NanoDrop 2000 (Thermo Fisher Scientific, Waltham, MA, USA). Complementary DNA (cDNA) was synthesized from 1 μg total RNA using the PrimeScript RT Reagent Kit (TaKaRa, Tokyo, Japan). qPCR was conducted on a StepOnePlus Real-Time PCR System (Applied Biosystems, Foster City, CA, USA) with SYBR Premix Ex Taq II (TaKaRa, Japan). Relative gene expression was calculated via the 2^-ΔΔCt^ method, with GAPDH as the internal reference.

### RNA sequencing and bioinformatics analysis

Total RNA was extracted from si-BUB1 and si-NC transfected A549 cells. RNA sequencing was performed by Novogene (Beijing, China) using the Illumina HiSeq X Ten platform. Gene Ontology (GO) and Kyoto Encyclopedia of Genes and Genomes (KEGG) pathway enrichment analyses were conducted using the DAVID database.

### Transmission electron microscopy

Cells were fixed with 2.5% glutaraldehyde (Beijing Solarbio) overnight, post-fixed with 1% osmium tetroxide for 1 h, dehydrated with gradient ethanol and embedded in epoxy resin. Ultrathin sections (50 nm) were stained with uranyl acetate and lead citrate, then observed under a TEM (Hitachi H-7650, Tokyo, Japan).

### Detection of intracellular Fe²^+^, MDA levels, and GSH activity

Intracellular Fe²^+^ levels were measured using an Iron Assay Kit (Beyotime, Shanghai, China). MDA levels and GSH activity were detected with MDA Assay Kit and GSH Assay Kit (Beyotime, Shanghai, China), respectively. Absorbance was measured with a microplate reader, and concentrations/activities were calculated based on standard curves.

### Luciferase reporter assay

The GPX4​promoter fragment was generated by PCR​and inserted into pGL3-basic. LUAD cells (3 × 10^4^/well, 24-well, triplicate) were transfected with the PGL3-GPX4/promoter reporter plus Renilla, then STAT3 or mock at 24 h. Firefly/Renilla activities were assayed with the Dual-Luciferase reporter kit.

### Co-immunoprecipitation assay

Cell lysates were prepared by resuspending samples in RIPA buffer, and clarified by centrifugation at 13,000 rpm for 20 min at 4 °C. The supernatant and the primary antibody were incubated overnight on a rotary mixer at 4 °C. Then, Protein G magnetic beads (HY-K0202, MCE) were added and the incubation continued. Finally, the binding protein was eluted and analyzed.

### Lipid ROS assay

The prepared cells were evenly spread on a 12-well plate and 10 μM BODIPY-581/591C11 (D3861, Thermo Fisher Scientific) was added. Incubation was carried out at 4 °C and 5% CO_2_ for 30 minutes. After washing twice with PBS, the ROS level was analyzed using a CytoFLEX cell analyzer.

### Immunofluorescence staining

Cells grown on coverslips or paraffin-embedded tumor sections were fixed with 4% paraformaldehyde, permeabilized with 0.1% Triton X-100, and blocked with 5% BSA. Samples were incubated overnight at 4 °C with primary antibodies against STAT3 and p-STAT3, then with Alexa Fluor 488 or 594-conjugated secondary antibodies (Invitrogen, USA) for 1 h at room temperature. Nuclei were stained with DAPI for 5 min. Images were captured using a confocal laser scanning microscope (Leica TCS SP8, Wetzlar, Germany), and fluorescence intensity was quantified with ImageJ software.

### Western blot analysis

Total protein was extracted using RIPA lysis buffer (Beyotime, Shanghai, China). Protein concentration was measured with a BCA Protein Assay Kit (Thermo Fisher Scientific, USA). Equal amounts of protein (30 μg) were separated by 10% SDS-PAGE and transferred to PVDF membranes (Millipore, Billerica, MA, USA). Membranes were blocked with 5% non-fat milk for 2 h at room temperature, then incubated overnight at 4 °C with primary antibodies against BUB1 (ab195268; 1:800; Abcam; USA), STAT3(ab68153; 1:1000; Abcam; USA), p-STAT3(ab76315; 1:800; Abcam; USA), GPX4 (ab125066; 1:1000; Abcam; USA) and β-actin (#21338; 1:5000; Signalway Antibody). After washing with TBST, membranes were incubated with horseradish peroxidase (HRP)-conjugated secondary antibodies (Bioss USA) for 1 h. Protein bands were visualized using an ECL Chemiluminescence Kit (Millipore, USA) and quantified with ImageJ software (v1.8.0).

### Animal experiments

BALB/c nude mice (4–6 weeks old) were purchased from Guangdong Provincial Medical Laboratory Animal Center. A549 cells transfected with sh-BUB1 or sh-NC (1×10^7^ cells/mouse) were subcutaneously injected into the right flank of nude mice. Tumor volume was measured every 3 days using a caliper (volume = (length × width²)/2). Four weeks post-injection, mice were euthanized by dislocating their cervical vertebrae under isoflurane anesthesia (4% isoflurane inhalation, followed by 2% isoflurane inhalation for anesthesia maintenance), and their tissue samples were then collected. Tumors were excised, weighed, and stored at −80 °C for further analysis. Animal experiments were approved by the Ethical Committee of the Laboratory Animal Center of Jinan University, Guangzhou, China.

### Statistical analysis

Data are presented as mean ± standard deviation (SD) from at least three independent experiments. Statistical analyses were performed using SPSS 22.0 software (IBM, Armonk, NY, USA) and GraphPad Prism 8.0 software (GraphPad Software, San Diego, CA, USA). Differences between groups were analyzed by Student’s t-test or one-way ANOVA followed by Tukey’s *post-hoc* test. P < 0.05 was considered statistically significant.

## Results

### BUB1 is overexpressed in LUAD and correlates with poor prognosis

**Figure 1:**
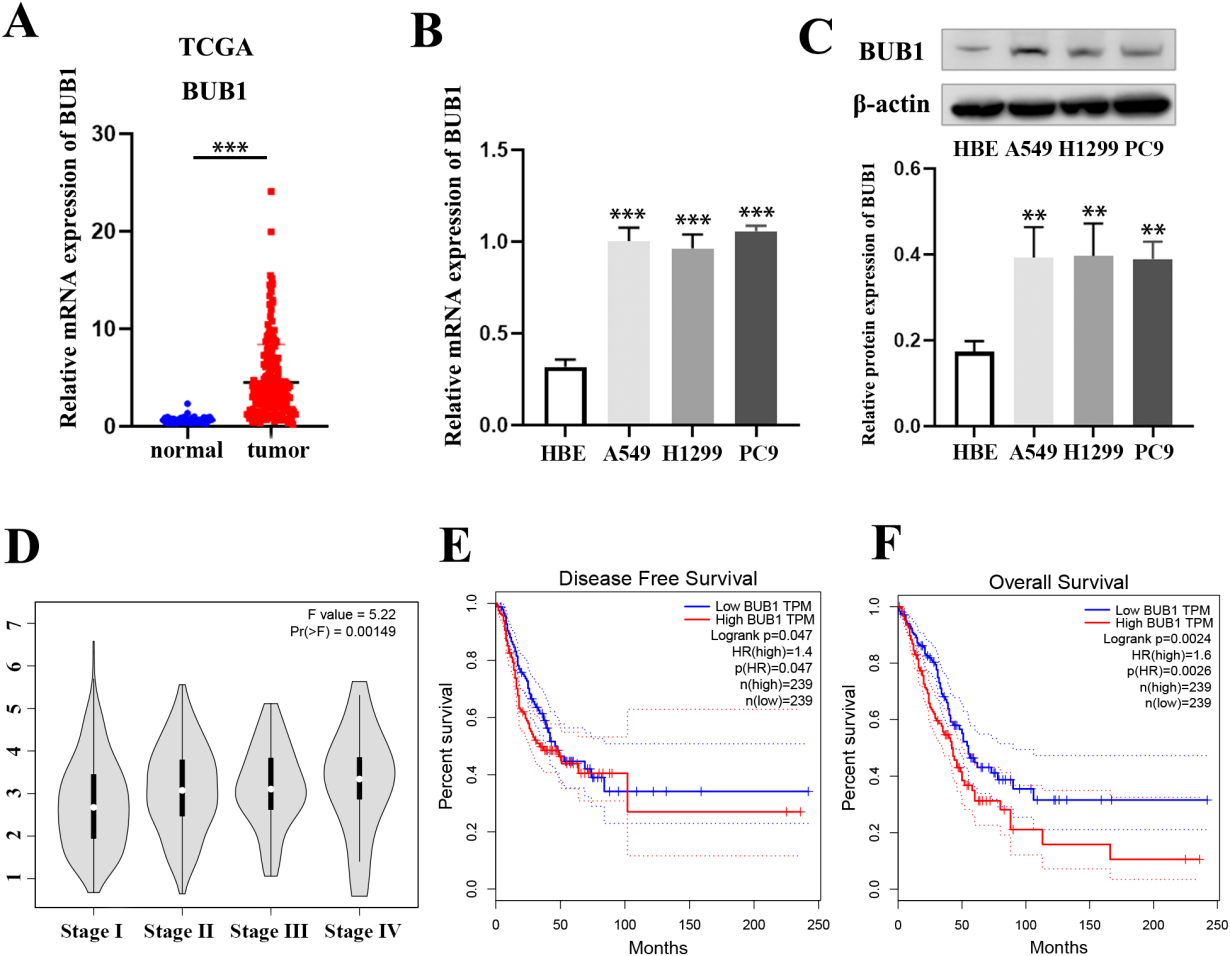
The expression and clinical association of BUB1 in LUAD. **(A)** Bioinformatics analysis of BUB1 expression in LUAD tumor tissues and matched normal tissues using the TCGA database. **(B)** RT-qPCR analysis of BUB1 mRNA levels. **(C)** Western blot analysis of BUB1 protein expression. **(D)** Association analysis of BUB1 expression and clinical stage of LUAD. **(E, F)** Kaplan-Meier survival curves showing disease-free survival (DFS, E) and overall survival (OS, F) of LUAD patients stratified by BUB1 expression levels (high vs. low) based on TCGA data. **P < 0.01 and ***P < 0.001.

Bioinformatics analysis using the TCGA database revealed significantly elevated BUB1 expression in LUAD tumor tissues relative to matched normal tissues (**Figure 1A**). This finding was further validated at the transcript level, as RT-qPCR demonstrated marked upregulation of BUB1 mRNA in LUAD cell lines (A549, H1299 and PC9) compared to the normal bronchial epithelial cell line HBE (**Figure 1B**). Consistently, western blot analysis confirmed a substantial increase in BUB1 protein abundance in these LUAD cell lines relative to HBE cells (**Figure 1C**). The association analysis between BUB1 expression and the clinical stage of LUAD showed that BUB1 expression was positively correlated with the malignancy degree of LUAD, and the BUB1 expression was higher in patients with advanced stage (Stage III-IV) (**Figures 1D**). Survival analysis based on TCGA data further supported the clinical relevance of BUB1 dysregulation. Kaplan-Meier curves indicated that high BUB1 expression correlated with markedly reduced disease-free survival (DFS) and overall survival (OS) in LUAD patients (**Figures 1E, F**). Collectively, these data demonstrate that BUB1 is aberrantly overexpressed in LUAD and that elevated expression levels are strongly predictive of poor clinical outcomes.

### Functional characterization of BUB1 in LUAD cells

**Figure 2:**
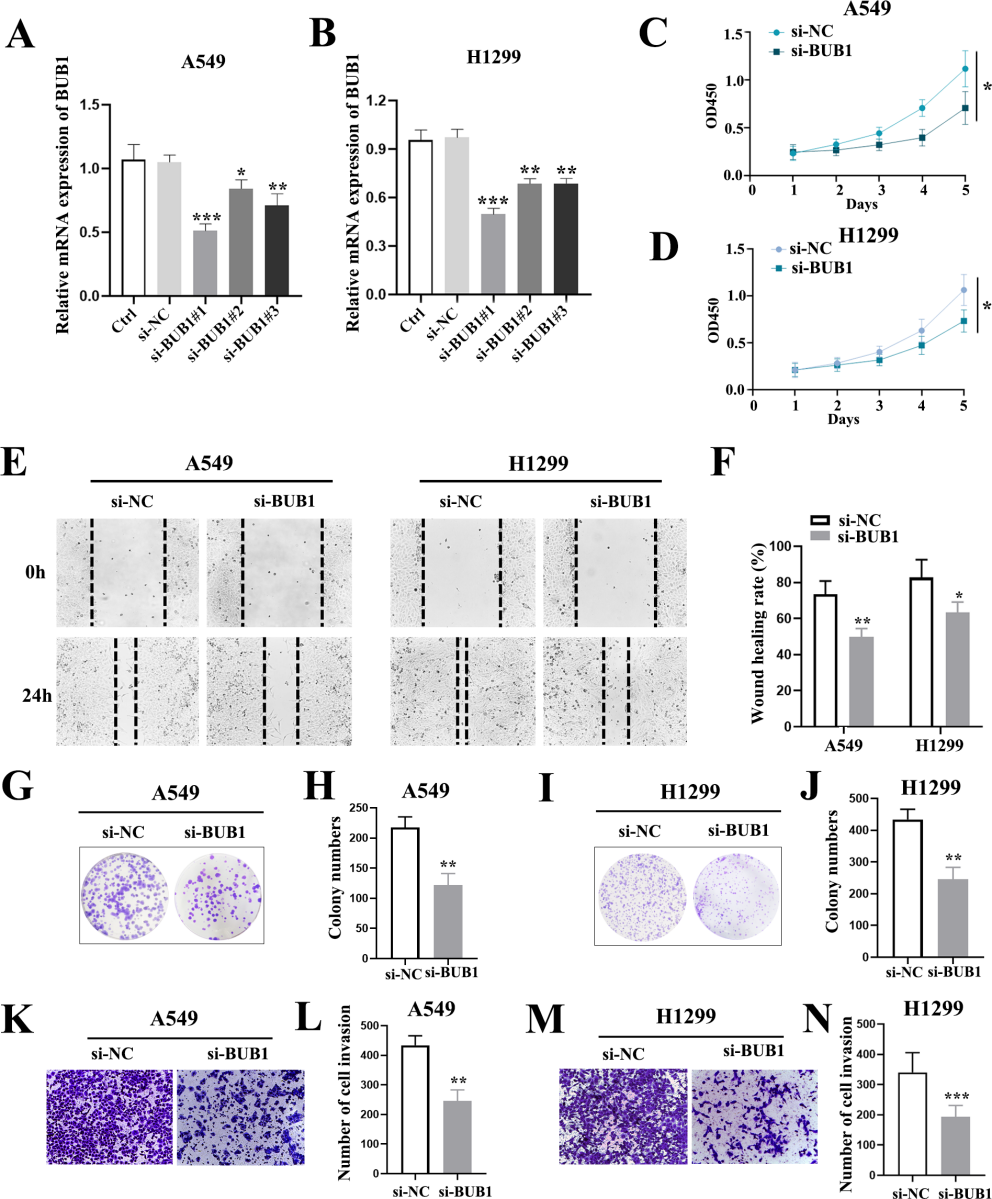
Silencing BUB1 inhibits the malignant phenotypes of LUAD cells. **(A, B)** RT-qPCR verification of BUB1 mRNA silencing efficiency in A549 **(A)** and H1299 **(B)** cells transfected with three si-BUB1 constructs (si-BUB1#1-3) or scramble control (si-NC). **(C, D)** CCK-8 assay showing cell viability (reflected by OD values) of A549 **(C)** and H1299 **(D)** cells after BUB1 silencing. **(E, F)** Wound healing assay images **(E)** and quantitative analysis **(F)** of A549 and H1299 cells at 0 h and 24 h post-scratching. **(G-N)** Colony formation assay **(G–J)** and Transwell invasion assay **(K–N)** showing the clonogenic potential and invasive capacity of LUAD cells after BUB1 knockdown. *P < 0.05, **P < 0.01 and ***P < 0.001.

To investigate the impact of BUB1 on LUAD cells, we systematically silenced BUB1 expression in A549 and H1299 cell lines using three distinct siRNA constructs (si-BUB1#1-3). RT-qPCR analysis confirmed significant BUB1 mRNA reduction in all siRNA groups compared to scramble controls (si-NC), with si-BUB1#1 demonstrating optimal suppression (**Figures 2A, B**). This siRNA was subsequently selected for functional studies based on its superior efficacy. In addition, we further found that BUB1 knockdown did not induce significant cytotoxicity in the normal HBE cells compared to the control group (**Supplementary Figure S1**).

As shown in **Figures 2C, D**, in both A549 and H1299 cells, the optical density (OD) values reflecting cell viability were notably lower in si-BUB1 groups than in si-NC groups. These data indicate that BUB1 knockdown suppresses the proliferative capacity of tumor cells. We further investigated the impact of BUB1 on cell migration via the wound healing assay. As depicted in **Figures 2E, F**, the wound closure in si-BUB1 groups was remarkably slower than that in si-NC groups at 24 hours after scratch wounding in both cell lines. Demonstrating that BUB1 silencing markedly suppressed the migratory capacity of A549 and H1299 cells. Furthermore, both colony formation and cell invasion assays indicated that BUB1 knockdown substantially inhibited the proliferative and invasive capabilities of LUAD cells relative to control cells (**Figures 2G–N**). These comprehensive findings establish BUB1 as a critical regulator of LUAD cell malignancy, with its silencing effectively targeting three hallmark cancer processes-uncontrolled proliferation, collective migration, and invasive potential.

### BUB1 suppresses ferroptosis in LUAD cells

**Figure 3:**
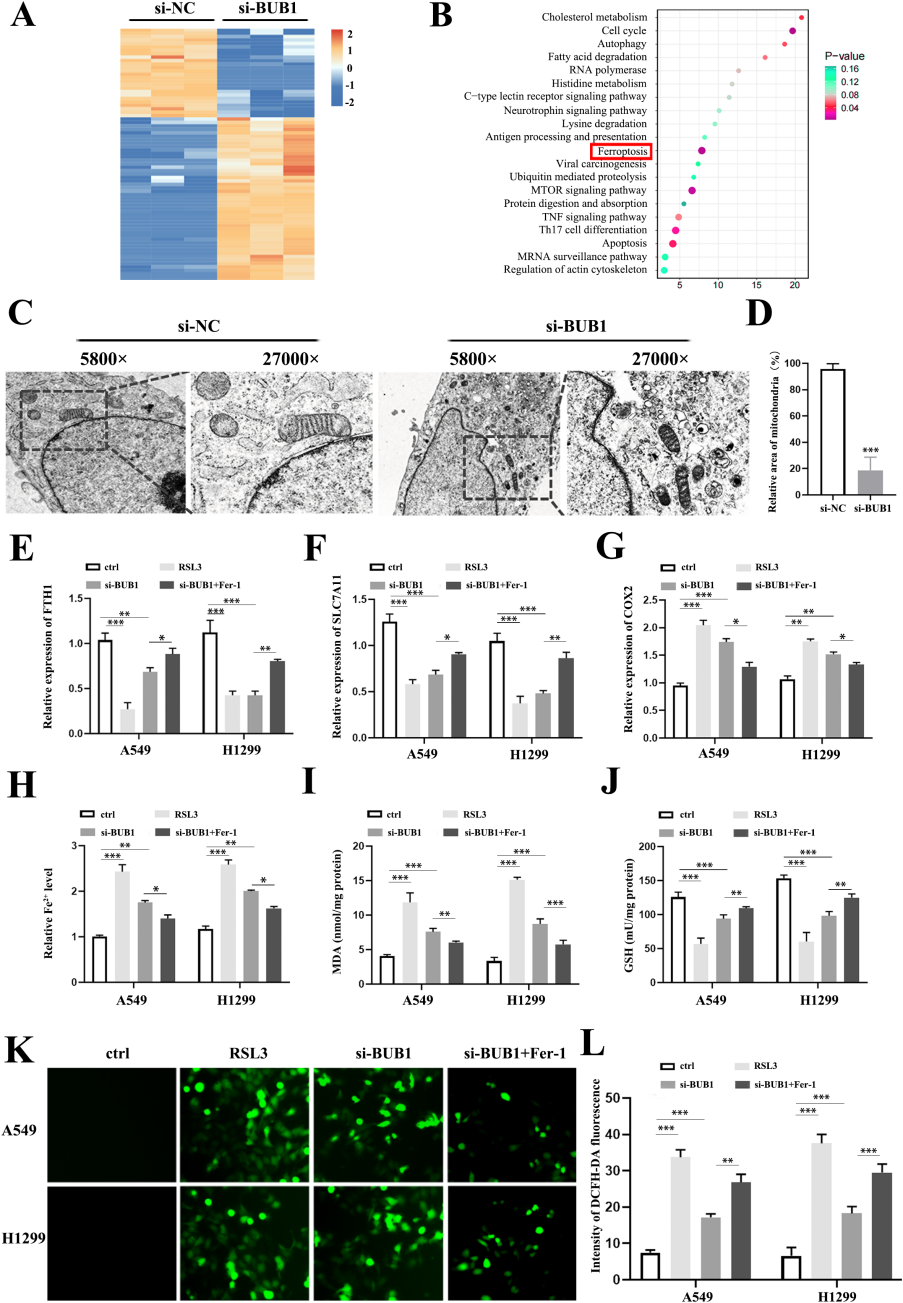
Knockdown induces ferroptosis in LUAD cells. **(A)** The heatmap of differentially expressed genes (DEGs) in BUB1-silenced A549 cells identified by RNA sequencing. **(B)** KEGG enrichment analysis showing DEGs enriched in ferroptosis-related pathways. **(C, D)** Transmission electron microscopy (TEM) images of LUAD cell. **(E–G)** RT-qPCR analysis of FTH1 **(E)**, SLC7A11 **(F)**, and COX2 **(G)** levels in A549/H1299 cells from four groups: ctrl, RSL3, si-BUB1, si-BUB1+Fer-1. **(H–J)** Quantitative detection of intracellular Fe²^+^ levels **(H)**, MDA content **(I)** and GSH activity **(J)** in each group. **(K, L)** lipid ROS measurement in each group. *P < 0.05, **P < 0.01 and ***P < 0.001.

Subsequently, RNA sequencing was conducted on BUB1-knockdown LUAD cells. This analysis identified a total of 293 differentially expressed genes (DEGs), with 174 being upregulated and 119 downregulated (**Figures 3A**). Enrichment analysis further indicated that ferroptosis was significantly enriched (**Figure 3B**). To exclude non-ferroptotic mechanisms, we inhibited necroptosis with Nec-1, autophagy with CQ, and apoptosis with Z-VAD in BUB1-silenced A549/H1299 cells. These treatments did not substantially reduce cell death, supporting a primary role for ferroptosis (**Supplementary Figure S2**). Based on these observations, we proposed the hypothesis that ferroptosis mediates the regulatory effect of BUB1 on LUAD cell progression.

To explore this, TEM was utilized to assess cellular ultrastructural changes. The BUB1-knockdown group displayed distinct ferroptosis-characteristic morphological features, including notably shrunken mitochondria, increased mitochondrial membrane density, and reduced mitochondrial cristae (**Figures 3C, D**). For functional validation, the ferroptosis inducer RSL3 and inhibitor Ferrostatin-1 (Fer-1) were used as experimental modulators, and cells were divided into four groups: ctrl, RSL3 treatment (RSL3), BUB1-knockdown (si-BUB1) and BUB1-knockdown plus Fer-1 (si-BUB1ckdownarac Ferroptosis-associated protein levels (FTH1, SLC7A11 and COX2) were assessed, and the results demonstrated that both RSL3 treatment and BUB1 silencing significantly decreased FTH1 and SLC7A11 expression, accompanied by an upregulation of COX2 in A549 and H1299 cells compared to the control group. Notably, these alterations were reversed by Fer-1 co-treatment (**Figures 3E–G**). Since intracellular iron accumulation is a hallmark of ferroptosis, we next quantified intracellular Fe^2+^ levels across all groups. Results showed that both RSL3 treatment and BUB1 silencing significantly increased intracellular iron concentrations in A549 and H1299 cells, while Fer-1 administration markedly attenuated this iron accumulation (**Figure 3H**). Malondialdehyde (MDA) and glutathione (GSH) are critical regulators of cellular redox homeostasis, and their levels undergo characteristic changes during ferroptosis. Compared to the control group, MDA levels were significantly elevated in the RSL3 and BUB1-silenced groups, and this increase was effectively abrogated by Fer-1 treatment (**Figure 3I**). Conversely, GSH activity was significantly reduced in both the RSL3 and BUB1-silenced groups, and this inhibitory effect was reversed by Fer-1 co-treatment (**Figure 3J**). The level of lipid-ROS was obviously reduced with in both the RSL3 and BUB1-silenced treatment, but restored by Fer-1 co-treatment (**Figures 3K, L**). Collectively, these findings confirm our hypothesis that BUB1 knockdown promotes ferroptosis in LUAD cells, mimicking the effect of the ferroptosis inducer RSL3.

### BUB1 affects STAT3/GPX4 pathway

**Figure 4:**
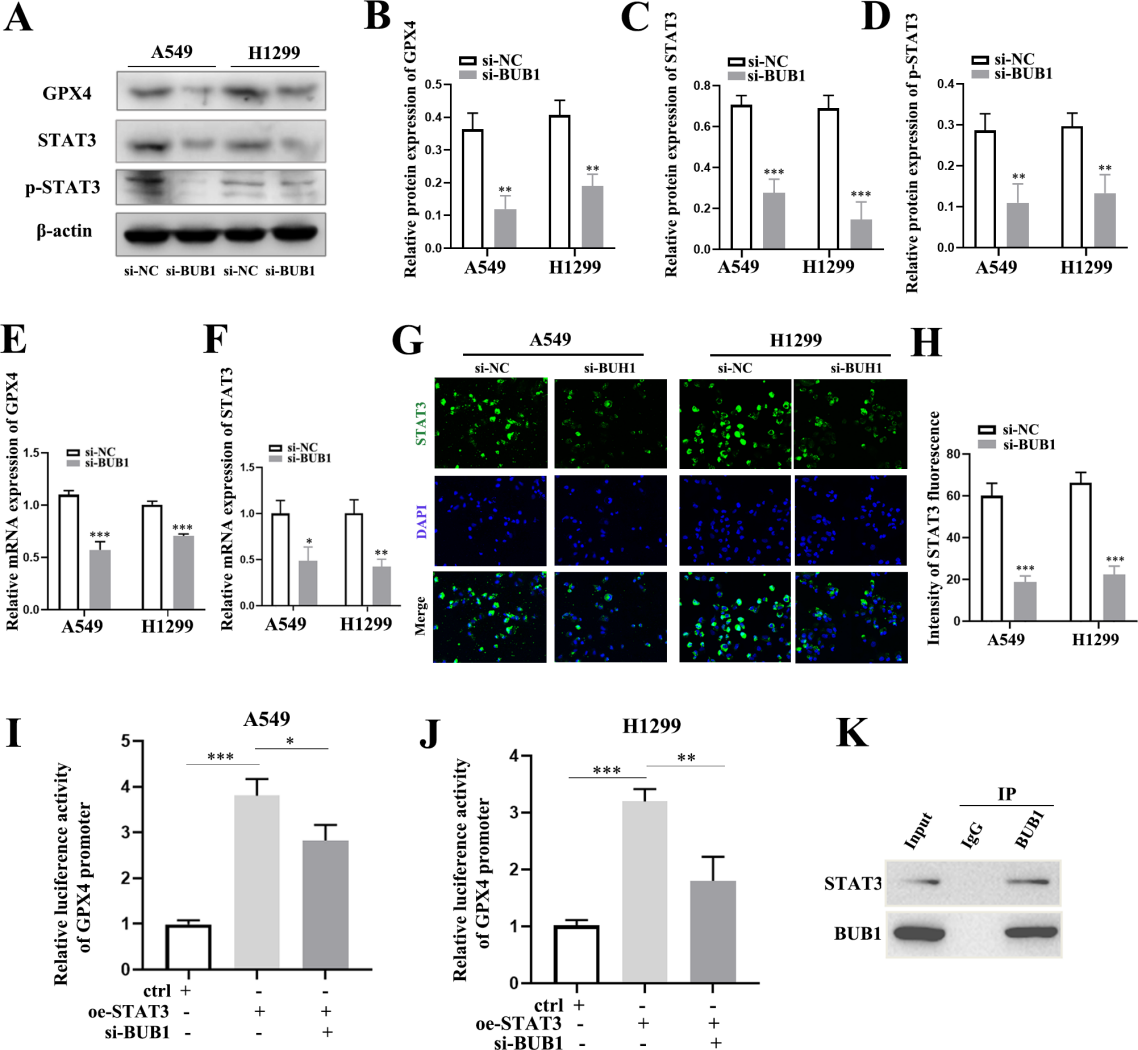
BUB1 regulates GPX4 expression via STAT3 in LUAD cells. **(A–D)** Western blot analysis of GPX4, STAT3 and p-STAT3 protein levels in A549 and H1299 cells after BUB1 silencing. **(E)** RT-qPCR analysis of GPX4 mRNA level in A549 and H1299 cells. **(F)** RT-qPCR analysis of STAT3 mRNA level in A549 and H1299 cells. **(G, H)** Immunofluorescence staining of STAT3 in A549 and H1299 cells (si-NC vs. si-BUB1). Nuclei were stained with DAPI. Scale bar: 50 μm. **(I, J)** Luciferase reporter assays in A549 and H1299 cells. **(K)** Co-immunoprecipitation assay in A549 and H1299 cells. *P < 0.05, **P < 0.01 and ***P < 0.001.

To elucidate the molecular mechanism by which BUB1 knockdown may contribute to the induction of ferroptosis, we focused on glutathione peroxidase 4 (GPX4), a critical enzyme maintaining lipid bilayer integrity through lipid peroxidation detoxification (15). Western blot analysis revealed significant downregulation of GPX4 protein levels following BUB1 knockdown in both A549 and H1299 cells (**Figures 4A, B**), accompanied by reduced GPX4 mRNA expression (**Figure 4E**). Given that previous studies have reported GPX4 to be regulated by multiple molecular factors, including STAT3, we hypothesized that BUB1 might influence GPX4 function via modulation of STAT3. To test this, we conducted additional western blot experiment and observed that BUB1 silencing potently suppressed STAT3 protein expression and its phosphorylation status (**Figures 4A–D**). Furthermore, BUB1 suppressed the transcription of STAT3 (**Figure 4F**) and reduced its levels in both the cytoplasm and nucleus (**Figures 4G, H**), as shown by RT-qPCR and immunofluorescence staining. To dissect the effect of STAT3 on GPX4, dual luciferase and coimmunoprecipitation (CO-IP) were carried out. A luciferase assay reveled that STAT3 activated the GPX4 promoter in A549/H1299 cells, and BUB1 knockout negated this activation (**Figures 4I, J**). Co-IP was designed to confirm a high degree of integration between STAT3 and GPX4 (**Figure 4K**). In summary, these findings suggest that BUB1 knockdown suppresses GPX4 transcription by downregulating STAT3 expression, thereby promoting ferroptosis in LUAD cells.

### STAT3 overexpression partially reversed the suppression of malignant phenotypes in LUAD cells induced by BUB1 knockdown

**Figure 5:**
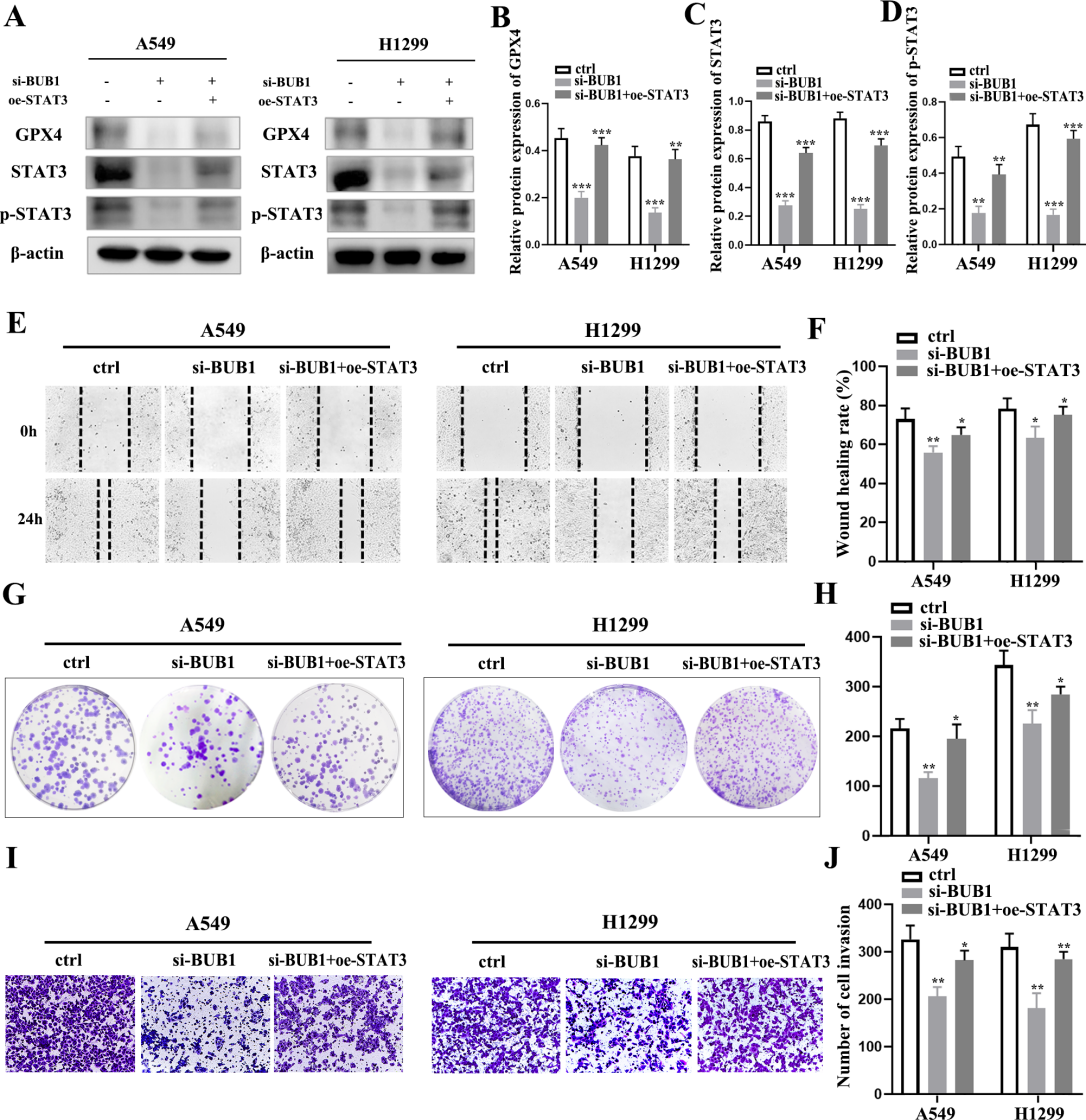
STAT3 overexpression reverses BUB1 silencing-induced suppression of LUAD cell malignancy. **(A–D)** Western blot analysis of GPX4, STAT3 and p-STAT3 protein levels in A549 and H1299 cells receiving different treatments (ctrl, si-BUB1 and si-BUB1+oe- STAT3). **(E, F)** Wound healing assay and quantitative analysis of migratory capacities of LUAD cells in each group. **(G–J)** Colony formation assay **(G, H)** and Transwell invasion assay **(I, J)** showing clonogenic potential and invasive capacities of LUAD cells. *P < 0.05, **P < 0.01 and ***P < 0.001.

To investigate whether BUB1 regulates the progression of LUAD through the STAT3/GPX4 axis, we overexpressed STAT3 in A549 and H1299 cells and subsequently subjected them to BUB1 knockdown. An analysis of GPX4, STAT3 and p-STAT3 levels showed that the BUB1 silencing-induced reduction in GPX4, STAT3 and p-STAT3expression was partially rescued by STAT3 overexpression (**Figures 5A–D**). The same intervention was then used to observe the malignant phenotypes of the two LUAD cells. The results indicated that STAT3 overexpression partially mitigated the migratory capacity induced by BUB1 silencing in both cell lines, as shown in **Figures 5E, F**. Likewise, STAT3 overexpression partially reversed the decrease in clonogenic potential (**Figures 5G, H**) and invasive ability (**Figures 5I, J**) caused by BUB1 knockdown in LUAD cells.

### Mechanistic insight into BUB1 silencing-induced ferroptosis via STAT3/GPX4 regulation

**Figure 6:**
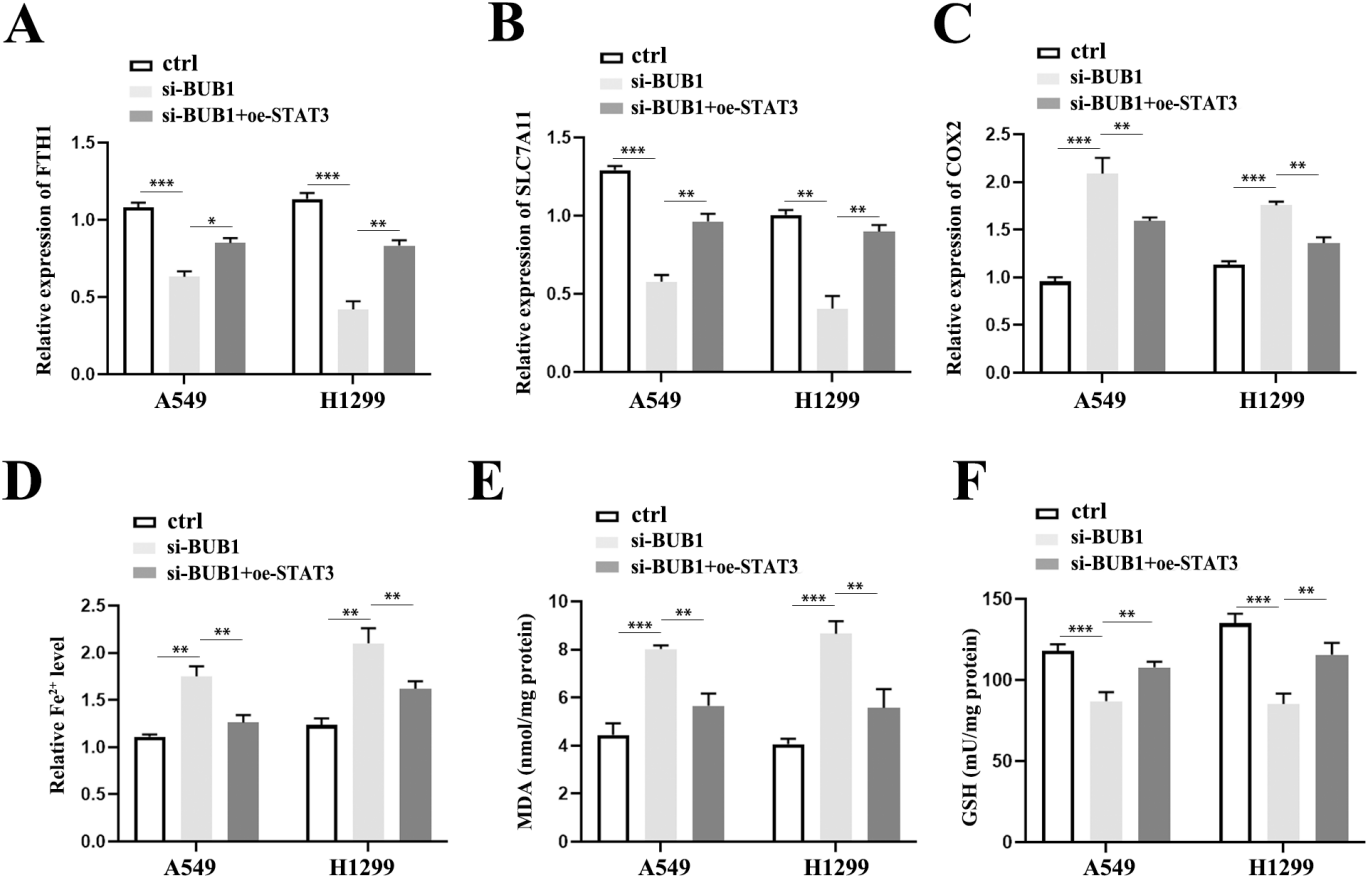
STAT3/GPX4 axis mediates BUB1 silencing-induced ferroptosis. **(A–C)** RT-qPCR analysis of FTH1 **(A)**, SLC7A11 **(B)**, and COX2 **(C)** levels in A549/H1299 cells from three groups: ctrl, si-BUB1 and si-BUB1+oe- STAT3. **(D–F)** Quantitative detection of intracellular Fe²^+^ levels **(D)**, MDA content **(E)** and GSH activity **(F)** in each group. *P < 0.05, **P < 0.01 and ***P < 0.001.

To investigate the involvement of the STAT3/GPX4 axis in ferroptosis, we overexpressed STAT3 and GPX4 in LUAD cells and evaluated its impact on ferroptosis-related responses. Compared with the si-BUB1-treated group, both STAT3 overexpression and GPX4 overexpression could counteract the effects of BUB1 silencing on the expression of key ferroptosis-associated genes, including FTH1 (**Figure 6A**, **Supplementary Figure S3A**), SLC7A11(**Figure 6B**, **Supplementary Figure S3B**), and COX2 (**Figure 6C**, **Supplementary Figure S3C**). We further assessed functional ferroptosis markers, including Fe^2+^, MDA and GSH activity. BUB1 knockdown led to a significant increase in Fe^2+^ levels, an effect that was markedly attenuated upon both STAT3 and GPX4 overexpression (**Figure 6D**, **Supplementary Figure S3D**). Similarly, the BUB1 silencing-induced elevation in MDA (**Figure 6E**, **Supplementary Figure S3E**) and reduction in GSH activity (**Figure 6F**, **Supplementary Figure S3F**) were both reversed by STAT3 and GPX4 overexpression. Collectively, these findings indicate that BUB1 silencing promotes ferroptosis in LUAD cells through modulation of the STAT3/GPX4 signaling pathway.

### Silencing of BUB1 benefits to treating LUAD *in vivo*

**Figure 7:**
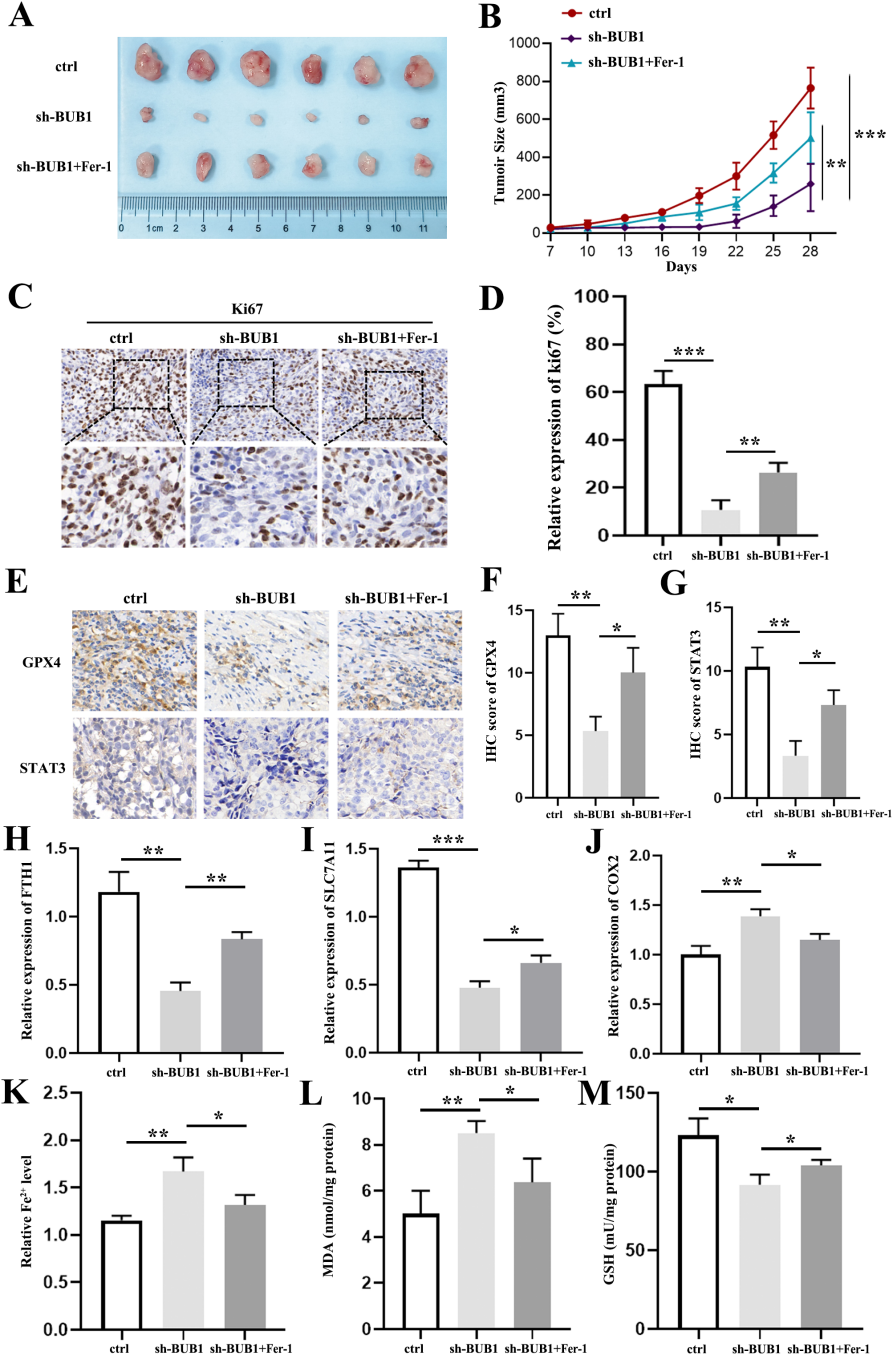
Silencing BUB1 inhibits LUAD tumor growth and modulates ferroptosis-related molecules *in vivo*. **(A, B)** Representative images of xenograft tumors **(A)**, tumor volume growth curves **(B)** in nude mice injected with si-BUB1 or si-BUB1+Fer-1 transfected A549 cells. **(C, D)** Immunohistochemical (IHC) staining of Ki67 in tumor sections **(C)** and quantitative analysis of staining intensity **(D)**. Scale bar: 50 μm. **(E–G)** Immunohistochemical (IHC) staining of GPX4 and STAT3 in tumor sections **(E)** and quantitative analysis of staining intensity **(F, G)**. Scale bar: 50 μm. **(H–J)** RT-qPCR analysis of FTH1 **(H)**, SLC7A11 **(I)**, and COX2 **(J)** mRNA levels in xenograft tissues. **(K–M)** Quantitative detection of intracellular Fe²^+^ levels **(K)**, MDA content **(L)** and GSH activity **(M)** in xenograft tissues. *P < 0.05, **P < 0.01 and ***P < 0.001.

To further dissect the role of BUB1 in a xenograft tumor mouse model, we found that BUB1 knockdown significantly reduced tumor volume (**Figures 7A, B**) compared to the control group, and Fer-1 could abolished this effect. Subsequent molecular analysis revealed that the proliferation marker Ki67, GPX4 and STAT3 were all lower in the BUB1 knockdown group than in the control (**Figures 7C–G**). Additionally, analysis of ferroptosis-related gene expression demonstrated that BUB1 knockdown led to downregulated FTH1 (**Figure 7H**) and SLC7A11 (**Figure 7I**) expression while increasing COX2 (**Figure 7J**) expression, these alterations were reversed by Fer-1 co-treatment, which were consistent with our *in vitro* results. Analysis of functional ferroptosis markers activity reveled that BUB1 silencing-induced elevation in Fe^2+^ and MDA levels (**Figures 7K, L**) while reduction in GSH activity (**Figure 7M**), these alterations were reversed by Fer-1 co-treatment, which were also consistent with our *in vitro* results. Collectively, these data confirm that BUB1 silencing not only suppresses LUAD growth but also modulates the expression of genes involved in ferroptosis.

## Discussion

Lung adenocarcinoma remains the leading cause of cancer-related mortality globally, primarily due to limited therapeutic advancements driven by insufficient specific molecular targets and incomplete elucidation of its pathogenic mechanisms (16, 17). Herein, we present systematic evidence indicating that BUB1 is aberrantly overexpressed in LUAD tissues and cell lines, with elevated expression strongly correlating with poor patient prognosis. Functional assays confirmed that silencing BUB1 inhibits LUAD cell proliferation, migration, and invasion while promoting ferroptosis both *in vitro* and *in vivo* through the activation of the STAT3/GPX4 signaling axis. These findings identify BUB1 as a promising prognostic biomarker and therapeutic target in LUAD, uncovering novel crosstalk between mitotic regulation and ferroptosis during lung cancer progression.

BUB1, a core component of the spindle assembly checkpoint, is classically recognized for its role in ensuring accurate chromosome segregation during mitosis. Dysregulation of BUB1 has been extensively documented in multiple malignancies, including breast and colorectal cancer, where it contributes to genomic instability and tumor progression (18). However, its clinical significance and functional role in LUAD remain undefined. To address this knowledge gap, we demonstrated for the first time that BUB1 expression is significantly upregulated in LUAD tissues and cell lines compared to normal controls. Importantly, Kaplan-Meier analysis of TCGA data revealed that high BUB1 expression is associated with markedly reduced disease-free survival (DFS) and overall survival (OS) in LUAD patients. This observation aligns with emerging evidence in pancreatic cancer (19), where elevated BUB1 expression similarly predicts unfavorable outcomes. Notably, siRNA-mediated BUB1 knockdown experiments further substantiated its oncogenic function, as BUB1 silencing significantly suppressed LUAD cell viability, colony formation, migration and invasion. These results suggest that BUB1 may serve as a conserved prognostic indicator across solid tumors, with our study specifically validating its relevance in LUAD.

Ferroptosis has emerged as a critical focus in tumor biology, recognized as an intrinsic tumor-suppressive mechanism that modulates tumor progression and therapeutic responsiveness (20). Inducing ferroptosis in cancer cells effectively suppresses proliferation and metastasis (21, 22). For example, hepatocellular carcinoma is inherently resistant to conventional chemotherapy, exhibits marked sensitivity to ferroptosis inducers, highlighting ferroptosis targeting as a viable therapeutic strategy (23). Similarly, ferroptosis induction significantly inhibits LUAD growth and metastatic potential (24–26). These findings suggest that ferroptosis-based therapeutic strategies may hold the key to overcoming current challenges in LUAD treatment.

Interestingly, in our study, RNA-seq analysis following BUB1 knockdown revealed significant enrichment of ferroptosis-associated pathways, prompting further investigation into the BUB1-ferroptosis regulatory axis. TEM observations confirmed that BUB1 silencing induced classic ferroptotic morphological changes in LUAD cells, including mitochondrial shrinkage and reduced cristae, which are hallmark features of ferroptosis that distinguish it from other modalities of cell death. Functional validation further demonstrated that BUB1 knockdown mimics the effects of the ferroptosis inducer RSL3, including reduced the expression of ferroptosis suppressors (FTH1 and SLC7A11), increased the expression of the ferroptosis marker COX2, elevated intracellular Fe²^+^ levels, and disrupted redox homeostasis, as evidenced by increased MDA levels and GSH activity. Critically, these effects were completely abrogated by the ferroptosis inhibitor Fer-1, confirming that BUB1 silencing specifically induces ferroptosis rather than exerting non-specific cytotoxicity. *In vivo* validation using a xenograft tumor model further corroborated these findings. BUB1 knockdown markedly attenuated tumor growth, as evidenced by reduced tumor volume, alongside modulation of ferroptosis-related genes and markers in tumor tissues. This represents a novel finding, as BUB1 has traditionally been studied exclusively in the context of mitosis and apoptosis. Our work is the first to demonstrate its role in suppressing ferroptosis in LUAD, indicating that its suppression not only impedes LUAD progression but also modulates ferroptosis within a physiologically relevant tumor microenvironment.

To delineate the molecular basis of BUB1-mediated ferroptosis regulation, we focused on GPX4, a master regulator of ferroptosis subject to transcriptional control by multiple factors (27), and its loss drives the buildup of lipid peroxides that critically dictate ferroptotic responses in cancer cells (28, 29). STAT3, a transcription factor implicated in ferroptosis (30), is downregulated by propofol, leading to reduced GPX4 expression and ferroptosis induction in CRC cells (31). Prior works have shown that STAT3 directly binds to the GPX4 promoter to enhance its expression, thereby suppressing ferroptosis in pancreatic cancer (14). Our study extends this regulatory axis to LUAD. We found that BUB1 knockdown significantly reduced GPX4 expression at both the protein and mRNA levels, concomitant with diminished STAT3 expression and phosphorylation. Immunofluorescence staining further confirmed that BUB1 silencing reduced STAT3 accumulation in both the cytoplasm and nucleus, a critical step for STAT3-mediated transcriptional regulation. Rescue experiments provided direct evidence for the STAT3/GPX4 axis: STAT3 and GPX4 overexpression partially rescued the BUB1 knockdown-induced malignant phenotype of LUAD cells. More importantly, STAT3 and GPX4 overexpression abrogated BUB1 silencing-mediated ferroptosis, normalizing the expression of FTH1, SLC7A11, and COX2, reducing intracellular Fe²^+^ and MDA levels, and restoring GSH activity. Collectively, these results collectively demonstrate that BUB1 regulates LUAD progression and ferroptosis by activating the STAT3/GPX4 axis. However, several limitations should be acknowledged. Firstly, the clinical validation relied primarily on TCGA dataset, which may not fully represent the heterogeneity of LUAD patients. Future studies with larger, multi-center cohorts and longitudinal follow-up are needed to validate BUB1 as a robust prognostic biomarker. Secondly, while our data suggest a role for BUB1 in regulating STAT3 activation, the precise mechanism remains to be fully elucidated. Future studies will employ pharmacological inhibitors of BUB1’s kinase domain or kinase-dead BUB1 mutants to directly test whether BUB1’s kinase activity is required for STAT3 phosphorylation and the subsequent suppression of ferroptosis. Thirdly, patient-derived organoids (PDOs) and orthotopic transplantation models should be employed to better recapitulate clinical scenarios. Addressing these gaps will not only strengthen the mechanistic understanding but also accelerate the translation of BUB1-targeted therapies for LUAD patients. These are the directions for our future research.

## Conclusion

In summary, our study identifies BUB1 as a significantly overexpressed gene in LUAD, with elevated expression levels strongly correlating with poor prognosis. Functional studies demonstrated that BUB1 silencing inhibits LUAD cell proliferation, migration, invasion, and tumor growth, while simultaneously inducing ferroptosis through the downregulation STAT3/GPX4 axis. These findings establish BUB1 as a prognostic biomarker and therapeutic target for LUAD, offering a novel ferroptosis-inducing treatment strategy.

## Data Availability

The data presented in the study are deposited in the OMIX repository, a publicly available repository. The accession number is OMIX014441 (BioProject: PRJCA055839).

## Supplementary material

Supplementary Figure 1 Silencing BUB1 has no effect on the normal HBE cell. CCK-8 assay showing cell viability (reflected by OD values) of normal HBE cell. ns, No statistical significance.

Supplementary Figure 2 The effect of BUB1 knockdown alone or combines with different cell death inhibitors on the cell viability of LUAD cells. **(A, D)** A549 and H1299 cells were treated with si-BUB1 with or without Nec-1, and cell viability was detected. **(B, E)** A549 and H1299 cells were treated with si-BUB1 with or without Z-VAD, and cell viability was detected. **(C, F)** A549 and H1299 cells were treated with si-BUB1 with or without CQ, and cell viability was detected. ns, No statistical significance.

Supplementary Figure 3 STAT3/GPX4 axis mediates BUB1 silencing-induced ferroptosis. **(A-C)** RT-qPCR analysis of FTH1 **(A)**, SLC7A11 **(B)**, and COX2 **(C)** levels in A549/H1299 cells from three groups: ctrl, si-BUB1 and si-BUB1+oe- GPX4. **(D-F)** Quantitative detection of intracellular Fe²^+^ levels **(D)**, MDA content **(E)** and GSH activity **(F)** in each group. **P *< 0.05, ***P *< 0.01 and ****P* < 0.001.
