# Supplementary figures and images for: BUB1 promotes lung adenocarcinoma progression by regulating STAT3/GPX4-mediated ferroptosis
Source: Front Oncol. 2026 Feb 2;15:1745238. doi: 10.3389/fonc.2025.1745238 (PMC12907760; doi:10.3389/fonc.2025.1745238)

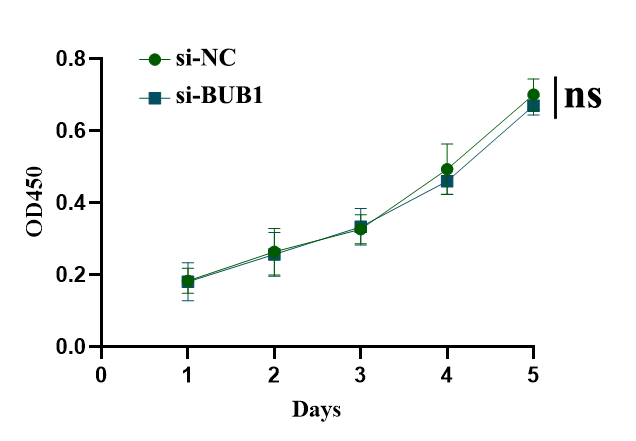

Supplement: Supplementary Figure 1 — Silencing BUB1 has no effect on the normal HBE cell. CCK-8 assay showing cell viability (reflected by OD values) of normal HBE cell. ns, No statistical significance. [file Image1.tif]

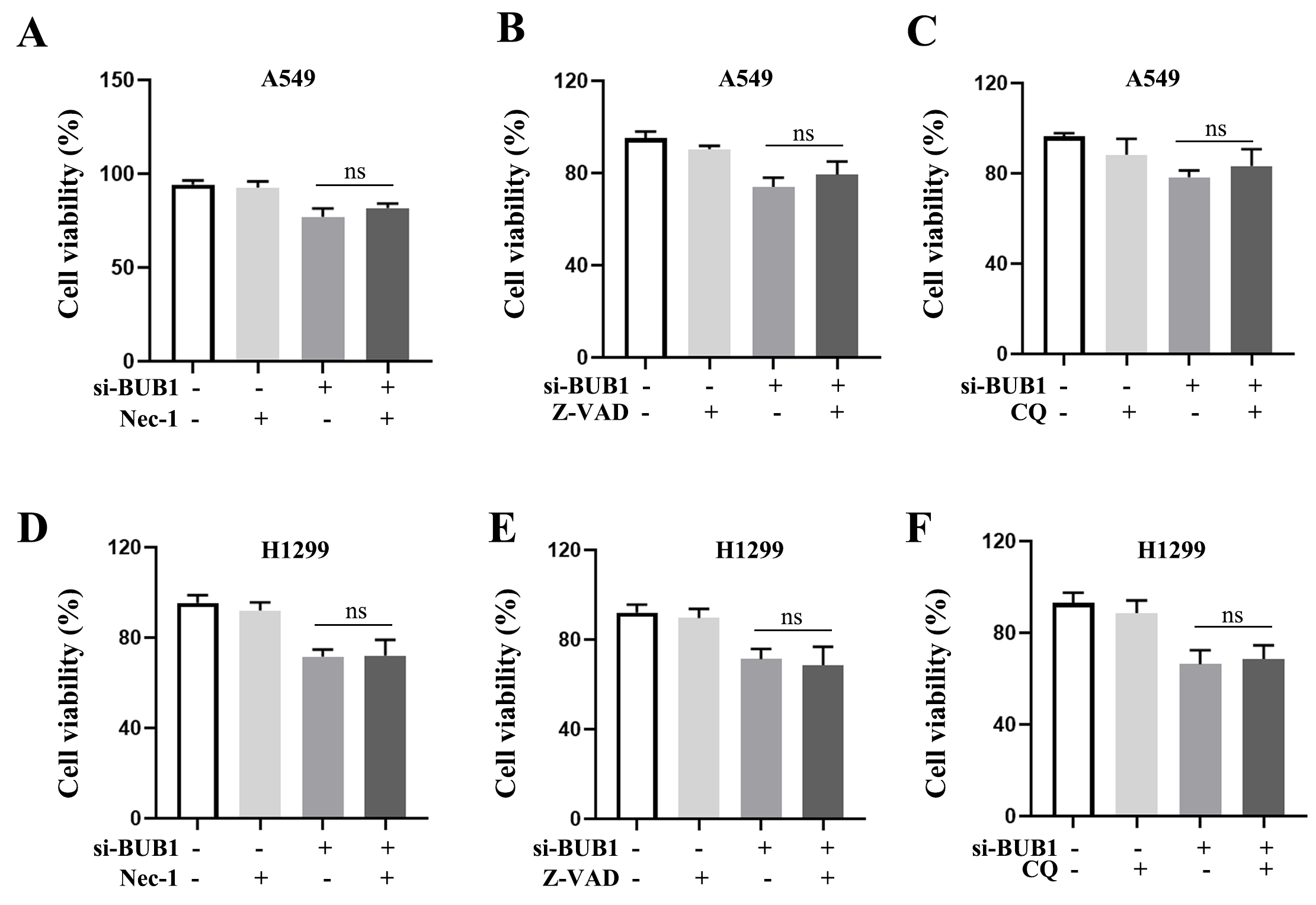

Supplement: Supplementary Figure 2 — The effect of BUB1 knockdown alone or combines with different cell death inhibitors on the cell viability of LUAD cells. (A, D) A549 and H1299 cells were treated with si-BUB1 with or without Nec-1, and cell viability was detected. (B, E) A549 and H1299 cells were treated with si-BUB1 with or without Z-VAD, and cell viability was detected. (C, F) A549 and H1299 cells were treated with si-BUB1 with or without CQ, and cell viability was detected. ns, No statistical significance. [file Image2.tif]

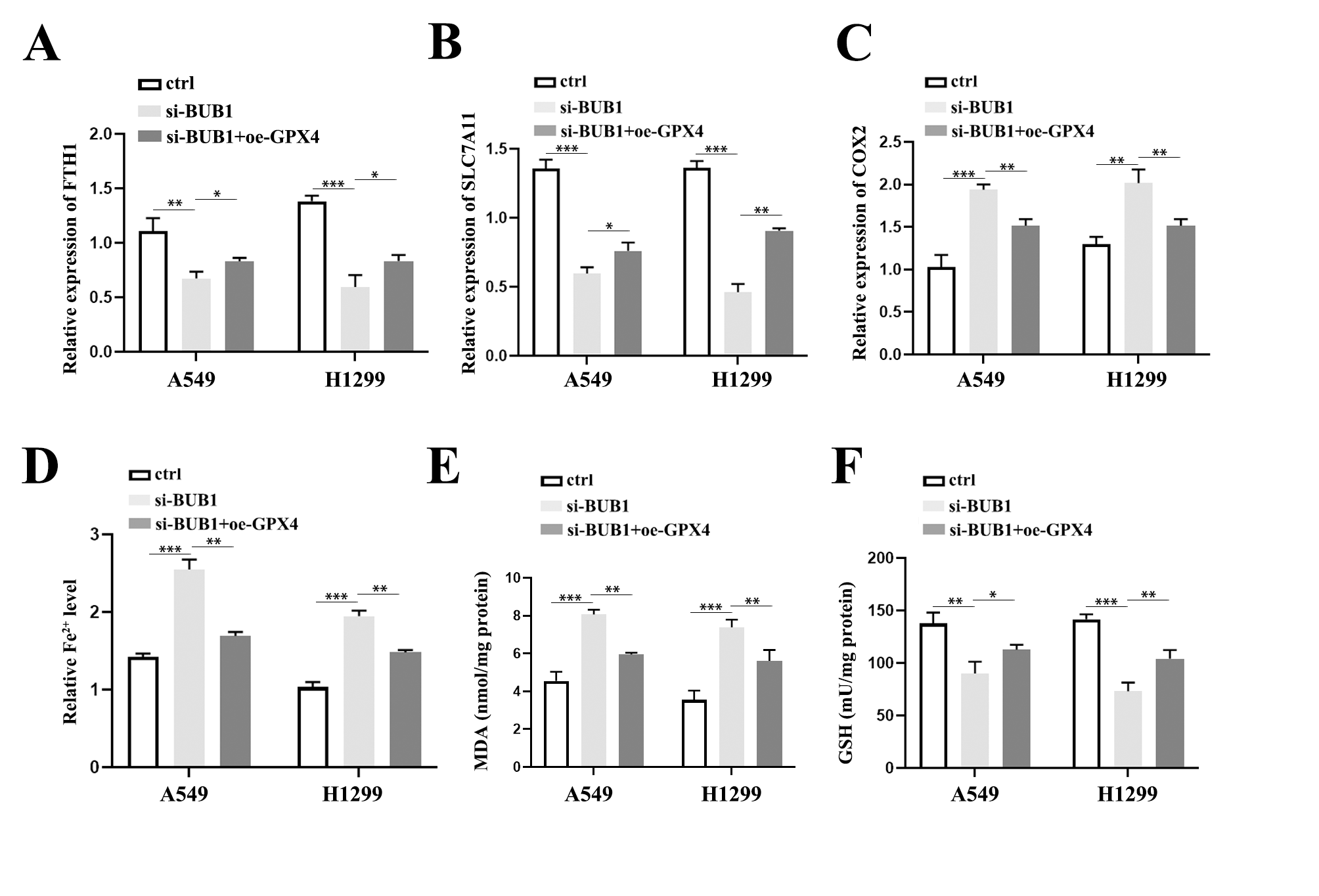

Supplement: Supplementary Figure 3 — STAT3/GPX4 axis mediates BUB1 silencing-induced ferroptosis. (A-C) RT-qPCR analysis of FTH1 (A), SLC7A11 (B), and COX2 (C) levels in A549/H1299 cells from three groups: ctrl, si-BUB1 and si-BUB1+oe- GPX4. (D-F) Quantitative detection of intracellular Fe²+ levels (D), MDA content (E) and GSH activity (F) in each group. *P < 0.05, **P < 0.01 and ***P < 0.001. [file Image3.tif]
